# Post-mortem evidence of a diverse distribution pattern of atherosclerosis in the South African population

**DOI:** 10.1038/s41598-022-15671-z

**Published:** 2022-07-05

**Authors:** Walter J. Janse van Rensburg

**Affiliations:** Human Molecular Biology Unit, School of Biomedical Sciences, Faculty of Health Sciences, University of the Free State, PO Box 339 (G2), Bloemfontein, Free State South Africa

**Keywords:** Cardiology, Cardiovascular diseases

## Abstract

Cardiovascular diseases (CVDs) are the number one cause of mortality worldwide. The disease profile of CVD varies considerably between different demographic groups and socioeconomic status. Atherosclerosis remains a major risk factor for CVD, and thus, believed to be a good indicator of the CVD profile in a population, yet little is known on its prevalence in sub-Saharan African populations. We aimed to determine the prevalence of atherosclerosis in a diverse South African population as found with post-mortem investigations. A retrospective file-audit was done on 10,240 forensic post-mortem reports done at a forensic pathology mortuary in South Africa, over 10-years. European descent males had the highest prevalence, with roughly one-quarter having coronary artery (CA) or large vessel (LV) atherosclerosis. European descent females followed closely, with one-fifth of the population having CA atherosclerosis and approximately a quarter having LV atherosclerosis. African descent males and females had a substantially lower prevalence in atherosclerosis for both CAs and LVs than European descendants. The mixed-ancestry population had a slightly higher prevalence of atherosclerosis in CAs and LVs than in the African population; however, it was still far lower than the European group. Some deviations in prevalence were noted within certain groups over the course of 10-years. The substantial difference in prevalence of atherosclerosis shows that in our region a diverse distribution pattern between ethnic groups and genders is present. However, follow-up studies are required to elucidate aetiological factors in cardiovascular health in our region.

## Introduction

Cardiovascular diseases (CVDs) are reportedly the number one cause of mortality worldwide. CVDs encompass coronary heart disease (CDH), cerebrovascular disease, peripheral artery disease, rheumatic heart disease, congenital heart disease, deep vein thrombosis and pulmonary embolism^1^. In South Africa, diseases of the circulatory system (ICD-10: I00-I99)^2^ has been reported as the leading cause of death in the country at 18.9% of all deaths^3^. These diseases include acute rheumatic fever, chronic rheumatic heart diseases, hypertensive diseases, ischemic heart diseases, pulmonary heart disease and diseases of pulmonary circulation, other forms of heart disease, cerebrovascular diseases, diseases of arteries, arterioles and capillaries, diseases of veins, lymphatic vessels and lymph nodes not elsewhere classified, and other and unspecified disorders of the circulatory system ^2^. However, one South African study reported significant variation in the CVD-related chronic disease mortality between different ethnic groups. When evaluating CVDs and other chronic diseases, the proportion of deaths caused by CVDs in African, European, mixed-ancestry, and Indian descent populations was 23.25%, 40.98%, 31.3%, and 51.79%, respectively ^4^. Furthermore, it has been reported that the prevalence of CVD is inversely proportional to a region’s socioeconomic status, with a decline seen in CVD-related deaths in all high-income and some middle-income countries ^5^. Conversely, the global burden of disease of CVD in southern sub-Saharan Africa is seemingly increasing^6^. However, a recent study reported that in South Africa, the overall national prevalence of coronary heart disease (CHD) of 1.29% remains relatively low when compared to the 4.29% reported for stroke^7^.

It is also interesting to note that in a large global study, including 52 countries worldwide, it was found that people living in sub-Saharan Africa are more likely to have a premature myocardial infarction than anywhere else. Furthermore, they found that in South Africans of African descent, the risk to develop CVDs increases with years of formal education and income level; however, the opposite was observed in the mixed-ancestry and European descent populations in South Africa^8^ as well as in a multi-ethnic study in America^9^.

It was found that South Africans of African descent was in an earlier stage of epidemiological transition regarding CVD^8^. The theory of epidemiological transition that Omran first described in 1971 investigates the multidimensional change in patterns of health and disease and the influence that the demographic, economic and sociological determinants and consequences in a population have on these patterns^10^. It has been proposed that socioeconomic status is possibly one of the essential role-players in CVD aetiology^11^. One global study found that mortality rates for ischaemic heart disease and stroke have declined between 1990 and 2010, however, steeper declines were observed for developed than for developing countries^12^. However, coronary heart disease is still a growing concern in low- and middle-income countries^13^.

CVD-related death rates have traditionally been lower among South Africans of African descent compared to the other ethnic populations, a steady increase in the prevalence of CVDs and the incidence of premature CVD-related deaths in both rural and urban communities and across the socioeconomic spectrum has been observed in this population group; therefore, socioeconomic status is not a stand-alone driving force behind CVD in this population^8^. Thus, it is important to note possible differences and changes in underlying or contributing physiological factors in the different ethnic groups. Therefore, in this study we aimed to identify the prevalence of atherosclerosis in our diverse population, to elucidate its role in the aetiology of CVD.

Atherosclerosis, a chronic immunoinflammatory and fibroproliferative disease of large and medium-sized arteries, is decidedly the most frequent cause of CVDs. Atherosclerotic plaques contain a large lipid-rich core covered with a thin fibrous cap containing few smooth muscle cells and many macrophages. These plaques may have signs of angiogenesis, inflammation of the adventitia, and outward vascular remodelling. Roughly three-quarters of all lethal coronary thrombotic events are triggered by plaque rupture^14^. Conversely, the weakening of blood vessel walls by atherosclerosis has been proposed as a potential risk factor in developing life-threatening LV aneurysms^15^. Therefore, determining atherosclerosis in different age and ethnic groups will greatly contribute to our understanding of the onset and progression of CVDs within our population. The reporting of atherosclerosis is routinely performed as part of comprehensive forensic post-mortem autopsies and serves as a valuable source to evaluate the prevalence of atherosclerosis in a given population. Many historical studies have used post-mortem autopsies to determine the prevalence of atherosclerosis in specific population groups^16–20^.

These studies transformed the basic understanding of the emergence and development of CVD in especially young individuals, by demonstrating anatomically that atherosclerosis affect these individuals without clinical evidence of heart disease. Subsequently, these publications contributed to widespread implementation of public health policies focussed on targeting risk factors associated with CVD^21^.

The most extensive study to date using this type of data set was performed to establish the prevalence of CA and aortic atherosclerosis in 3832 United States of America Armed Services servicemen and women, who died of combat or of unintentional injuries in support of Operations Enduring Freedom and Iraqi Freedom/New Dawn between October 2001 and August 2011. They reported a total prevalence of atherosclerosis at 12.1%, the prevalence of both CA and aortic atherosclerosis at 2.1%, the prevalence of only CA atherosclerosis at 8.5%, and the prevalence of only aortic atherosclerosis at 5.7%. Their population consisted mainly out of people of European descent (72.7%, non-Hispanic), with limited African descent (8.1%), Hispanic (11.1%) or Asian/Pacific Islander (3.4%) representation^21^. Therefore, their results have limited bearing on the majority of the South African population.

It has been reported that post-mortem macroscopic atherosclerotic lesions were significantly more common in a South African mixed-ancestry population than in South Africans of African descent (54% vs 31%). Unfortunately, this South African study only evaluated 147 individuals, comprising of 138 males and 11 females, of which 115 were of African descent, 31 mixed-ancestry, three of European descent, and one of Asian descent. They point out that their small sample size is not representative of the broader South African community and recommend that further studies are required into the cardiovascular health of South Africans^22^. Furthermore, their study was conducted in the Western Cape Province of South Africa that has a much different demographic distribution than the rest of South Africa. People of African descent make only 35.7% of the Western Cape population, in contrast to the 80.7% in the whole of South Africa and the 88.7% in the Free State Province^23^. The Western Cape also has the highest average life expectancy of all provinces in South Africa (males: 64.9-years, females: 70.3-years), versus the Free State Province who has the lowest average life expectancy (males: 55.5-years, females: 61.4-years)^24^. Consequently, more studies are indeed required to comprehensively investigate the state of the diverse South African population’s cardiovascular health.

Therefore, our study aimed to determine the prevalence of CA and LV atherosclerosis in a diverse population in the Free State Province of South African, as determined on macroscopic post-mortem autopsy evaluation.

## Methods

Permission to conduct this study was given by the Health Sciences Research Ethics committee of the University of the Free State (UFS-HSD2021/0843/2707) and the Free State Provincial Department of Health (FS_202106_015). All research was performed in accordance with the Declaration of Helsinki. We performed a retrospective file audit of all unnatural and unexplained deaths evaluated at the Free State Forensic Pathology Mortuary in Bloemfontein, South Africa, from January 2010 to December 2019 (10-years). Due to the retrospective nature of this study and that the data was collected from post-mortem reports of deceased individuals, with no additional testing being done on any tissue, informed consent was not applicable and was waived by the Health Sciences Research Ethics committee of the University of the Free State due to the retrospective nature of the study. All personal identifying information was removed from the data in accordance with the Protection of Personal Information Act 4 of 2013 of South Africa. During the routine performance of all comprehensive forensic post-mortem autopsies, the presence (or absence), location (CAs or LVs such as the aorta, carotid, pulmonary, iliac, femoral, and renal arteries), and severity of atherosclerosis, as well as the level of stenosis are noted in the reports. We classified the severity of atherosclerosis according to the criteria set out by Webber et al. The criteria are as follows: mild (only fatty streaking observed), moderate (10–49% vessels stenosis), and severe (≥ 50% vessel stenosis)^21^.

Ethnicity was taken as perceived by the reporting forensic pathologist based on phenotypical characterisations and interviews with family and acquaintances of the deceased and noted in the formal post-mortem report. According to Statistics South Africa ethnicity in the country is broadly grouped into four distinct populations namely black African, coloured, Indian/Asian and white^23^. The black African population in South Africa consists mainly of people speaking the traditionally Southern Bantho languages of Pedi, Sotho, Tswana, Swati, Venda, Tsonga, Ndebele, Xhosa, and Zulu^25^. The traditionally white populations in South Africa are generally accepted as descendants of the early European colonisers of mainly Dutch, German, French and English origin^26^. These skin-coloured based classifications are commonly seen as outdated and not appropriate for academic literature, thus in this article, we refer to the originally black South Africans as being of African descent, white or Caucasian South Africans of European descent, coloured South Africans of mixed-ancestry, and Indian/Asian only as Asian descent.

## Results

### Demographics

An overall 10,240 files were assessed for this study. Files that did not contain post-mortem reports, cases where only partial autopsies were performed, cases where an advanced stage of decomposition were present, and severe trauma cases where the presence or absence of atherosclerosis were unevaluatable, were excluded from further analysis. A total of 1296 files were excluded. Therefore, ultimately a total of 8944 post-mortem autopsy reports were evaluated for atherosclerosis. The ethnic distribution was similar to the national census reported ethnic distribution in the region, with the main ethnic group being people of African descent (83.6% vs 88.7%), followed in descending order by the European descent (11.8% vs 8.5%), mixed-ancestry (3.8% vs 2.5%), and Asian descent (0.8% vs 0.3%) groups^23^. Due to the very low numbers in the Asian descent group (n = 69), statistical analysis was not performed on their data. We also did not perform gender stratified analysis in the mixed-ancestry group due to the small population size (n = 336). Males were overrepresented in all groups (roughly 76% of the total population), deviating from the normal male to female gender distribution in the region of approximately 1:1. Considering our study population, consisting of people who died unnatural and unexplained deaths, this finding was in line with the described gender distribution in this group. As across all age groups, men proportionally have a much higher chance of having external causes of mortality (such as accidents, homicide and suicide) than women^3^.

### Prevalence of atherosclerosis 2010–2019

**Figure 1:**
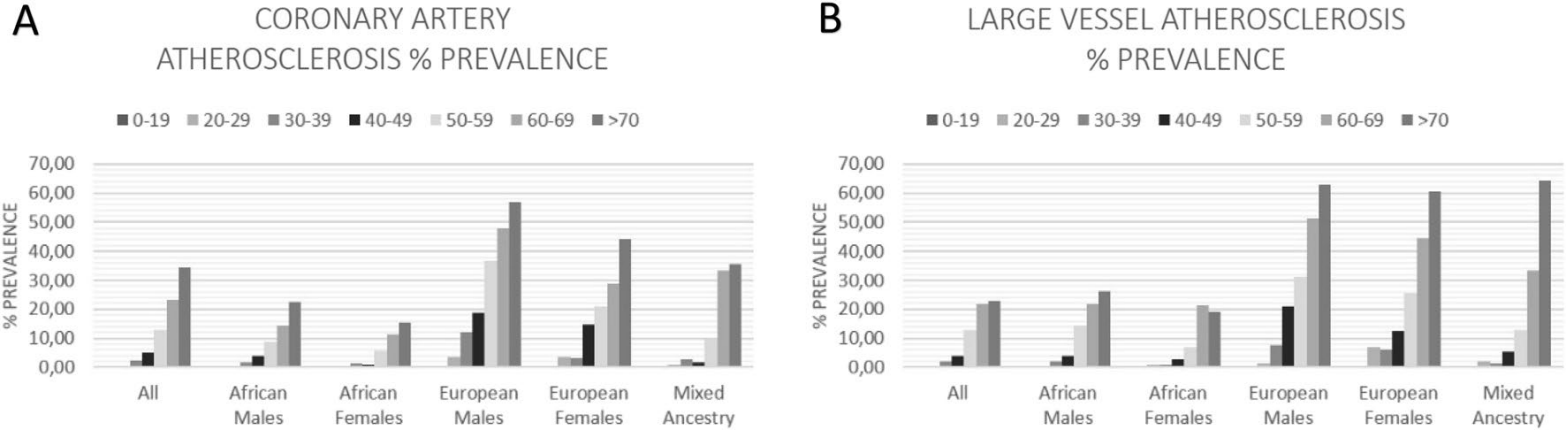
Prevalence of coronary artery (**A**) and large vessel (**B**) atherosclerosis in different population and age groups.

In this study we determined only the crude prevalence percentage of atherosclerosis against the unaffected individuals in our study population. Thus, incidence rates were not determined and estimates were not age or gender adjusted. The total prevalence of atherosclerosis was determined at 9.25% (n = 827/8944). The prevalence where both CA and LV atherosclerosis were seen was determined at 3.15% (n = 282/8944). The prevalence of a person with either CA atherosclerosis or LV atherosclerosis alone was determined at 2.42% (n = 216/8944) and 3.68% (n = 329/8944), respectively. The total prevalence of CA and LV atherosclerosis was found to be 5.57% (n = 498/8944) and 6.83% (n = 611/8944), respectively. The prevalence of both CA and LV atherosclerosis followed mostly an age-dependent trend increase for all population groups (Fig. 1). However, large differences were seen between ethnic groups and between genders within ethnic groups (Table 1).

**Table 1:** Prevalence of coronary artery and large vessel atherosclerosis per demographic group.

	African descent males	African descent females	European descent males	European descent females	Mixed ancestry
**Number of people with coronary artery atherosclerosis/number in group (prevalence %)**					
Totals	498/8944 (5.57%)				
	218/7476 (2.92%)		260/1057 (24.6%)		
	179/5772 (3.1%)	39/1702 (2.29%)	202/757 (26.68%)	58/300 (19.33%)	16/336 (4.76%)
Age groups					
0–19	0/716 (0%)	0/428 (0%)	0/39 (0%)	0/42 (0%)	0/64 (0%)
20–29	6/1640 (0.37%)	1/330 (0.3%)	5/136 (3.68%)	1/29 (3.45%)	1/90 (1.11%)
30–39	28/1581 (1.77%)	4/348 (1.15%)	13/107 (12.15%)	1/32 (3.13%)	2/71 (2.82%)
40–49	34/876 (3.88%)	3/269 (1.12%)	23/123 (18.7%)	7/48 (14.58%)	1/54 (1.85%)
50–59	50/579 (8.64%)	10/170 (5.88%)	52/141 (36.88%)	9/43 (20.93%)	3/31 (9.68%)
60–69	41/284 (14.44%)	9/79 (11.39%)	60/125 (48%)	13/45 (28.89%)	4/12 (33.33%)
> 70	20/88 (22.73%	12/78 (15.38%)	49/86 (56.98%)	27/61 (44.26%)	5/14 (35.71%)
**Number of people with large vessel atherosclerosis/number in group (prevalence %)**					
Totals	611/8944 (6.83%)				
	308/7476 (4.12%)		276/1057 (26.11%)		
	249/5772 (4.31%)	59/1702 (3.47%)	198/757 (26.16%)	78/300 (26%)	23/336 (6.85%)
Age groups					
0–19	1/716 (0.14%)	1/428 (0.23%)	0/39 (0%)	0/42 (0%)	0/64 (0%)
20–29	10/1640 (0.61%)	3/330 (0.91%)	2/136 (1.47%)	2/29 (6.9%)	2/90 (2.22%)
30–39	35/1581 (2.21%)	3/348 (0.86%)	8/107 (7.48%)	2/32 (6.25%)	1/71 (1.41%)
40–49	35/876 (4%)	8/269 (2.97%)	26/123 (21.14%)	6/48 (12.5%)	3/54 (5.56%)
50–59	83/579 (14.34%)	12/170 (7.06%)	44/141 (31.21%)	11/43 (25.58%)	4/31 (12.9%)
60–69	62/284 (21.83%)	17/79 (21.52%)	64/125 (51.20%)	20/45 (44.44%)	4/12 (33.33%)
> 70	23/88 (26.14%)	15/78 (19.23%)	54/86 (62.79%)	37/61 (60.66%)	9/14 (64.29%)

Males of European descent had the highest prevalences, with the prevalence of CA and LV atherosclerosis determined at 26.68% (n = 202/757) and 26.16% (n = 198/757), respectively. It equates to a substantial 3.79-fold and 2.83-fold increase in prevalence when compared to the total population. Moreover, 10.30% and 8.59% of all European descent males had severe CA or LV atherosclerosis, respectively. A substantial percentage of 56.98% (n = 49/86) of all European descent males over 70-years of age had CA atherosclerosis, with an even bigger proportion of 62.79% (n = 54/86) having LV atherosclerosis. Furthermore, severe atherosclerosis was seen in 24.42% (n = 21/86) and 32.56% (n = 28/86) of European males in this age group, for CAs and LVs, respectively. When taking the collective of all European males above 50-years of age, the prevalence of atherosclerosis for CAs and LVs were determined at 45.74% (n = 161/352) and 46.02% (n = 162/352). Even though European descent females weren’t as much affected as their male counterparts regarding CA atherosclerosis (19.33%, n = 58/300), they displayed a similar level of LV atherosclerosis (26.00%, n = 78/300). However, they still presented a much higher prevalence for both than the total population. It also results in a massive 2.47-fold and 2.81-fold increases in prevalence when compared to the total population. Conversely, African descent males and females only have prevalences of 3.10% (n = 179/5772) and 4.31% (n = 249/5772), and 2.29% (n = 39/1702) and 3.47% (n = 59/1702), for CA and LV atherosclerosis. People of mixed-ancestry remained relatively close to the total population’s statistics, with a prevalence of 4.76% (n = 16/36) and 6.85% (n = 23/336) for CA and LV atherosclerosis, respectively.

### Five-year progression analysis (2010–2014 vs 2015–2019)

**Figure 2:**
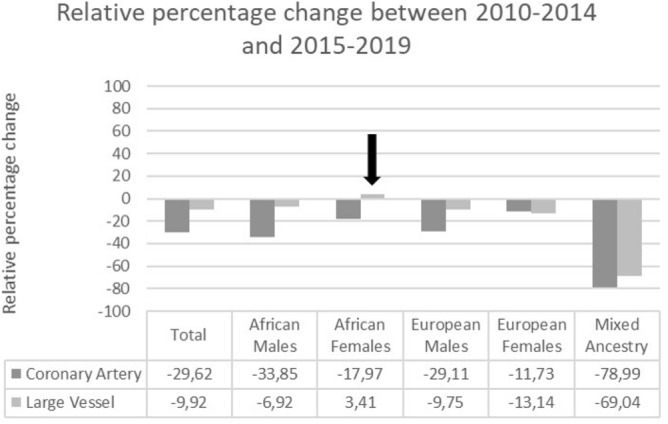
Relative percentage change in atherosclerosis prevalence over 5-years. The arrow indicates the only relative increase in prevalence over these periods.

We evaluated the prevalence after the first 5 years (2010–2014) and then again after the last 5 years (2015–2019). There was a 1.91% (29.62% relative) decrease in CA atherosclerosis for the total population and a 0.71% (9.92% relative) decrease in LV atherosclerosis between the two periods (Fig. 2). For CA atherosclerosis, all subgroups showed a reduction in prevalence between the two periods. The mixed-ancestry group showed the largest relative decrease (78.99%), and European descent females showed the lowest relative decrease (11.73%) of all the subgroups. However, dividing these groups into two time periods decreased the total numbers from 336 to 200 and 136 for the mixed-ancestry group, and from 300 to 151 and 149 for the European descent female group, thus, resulting in less statistical power from the data. The relative decreases for African descent males, African descent females, and European descent males were 33.85%, 17.97%, and 29.11%. For LVs, all subgroups, except females of African descent, showed a decrease in prevalence between the two periods. African descent females showed a slight relative increase of 3.41%. Again, the mixed-race group showed the biggest change, from 9.50% to 2.94%, which relates to a 69.04% relative decrease. However, due to the comparatively small population size, results from this group and the European descent female group (13.14% relative decrease) should be interpreted with caution. The relative declines for African descent males and European descent males were 6.62% and 9.75%. One noteworthy finding was that for both the African descent male and female groups in the 60 to 69-years age bracket, both CA and LV atherosclerosis prevalence increased between the two time periods. The prevalence of LV atherosclerosis increased with a substantial 8.76% (50.10% relative increase) in African descent males and 8.14% (46.52% relative increase) in African descent females.

## Discussion

The current study was the most extensive post-mortem study to date to determine the prevalence of atherosclerosis in CAs and LVs. The ethnic distribution of our study population was comparable to the general ethnic distribution in the diverse population of the Free State Province in South Africa^23^. The overall prevalence of atherosclerosis of 9.25% is lower than the overall prevalence of 12.1%, as seen in the most recent study in an American population. However, due to the big difference in ethnic distribution between their study population and ours (majority European descendants—72.73% vs majority African descendants—83.59%), the data should rather be compared according to the same ethnic groups. The whole African descent group had a much lower prevalence of overall atherosclerosis in the South African population (5.74%, n = 429/7476) when compared to the African-American population (12.86%, n = 40/311).

Conversely, the European descendants in South Africa had a substantially higher prevalence of total atherosclerosis (34.53%, n = 365/1057) when compared to their American counterparts (11.77%, n = 328/2787). However, it must also be noted that the American study consisted of mainly young people under the age of 40. The prevalence for overall atherosclerosis substantially increases in their study to 45.86% (n = 72/157) when looking at people over 40-years of age. When evaluating all European descendants over 40-years of age in our population, the prevalence of 50.00% (n = 336/672) was very similar to the overall American results in that age group. The overall prevalence for the African descendants in our study remained consistently lower (14.20%, n = 344/2423) than the European descendant and total American populations, even at the increased age groups^21^. An important limitation to note of both our study and the American study of Webber et al.^21^ was that males were heavily overrepresented in both studies, representing 77.21% and 98.25% of the two study populations, respectively. Therefore, evaluating each gender group on its own may be more representative of the specific populations.

As most of the population in the Webber et al. study consisted of males of European descent between roughly the ages of 20 and 40-years^21^, we decided to compare their data to our European descent male population in that age group. Somewhat predictably, these populations showed a comparable prevalence of total atherosclerosis (South African Europeans—9.88% vs American population—10.66%). When only evaluating atherosclerosis in CAs in this age group, the prevalence in the European male population (11.20%) was substantially lower than reported in earlier American studies between 1953 and 1993 that reported the prevalence of CA atherosclerosis of between 45% and 78.3% in mainly European descent young males^16–18^. Our findings in our European descent population correlating with the latest American data is not unexpected, taken that the decreasing trend in CA atherosclerosis prevalence in younger people has been previously described^19,27^.

According to Statistics South Africa, there has been a steady increase in the proportion of deaths caused by diseases of the circulatory system from 2010 to 2018. The percentage of deaths increased from 15.2 to 18.9% in this time. In the Free State Province, the proportion increased from 14.98 to 19.22% during the same time. Countrywide the proportion of deaths attributed to some of the specific causes increased steadily from 2010 to 2018 as follows: hypertensive diseases (2.0% to 3.1%), ischaemic heart diseases (2.5% to 3.2%), other forms of heart disease (4.2% to 4.6%), and cerebrovascular diseases (3.6% to 4.2%)^3,28–30^. These steady increases in occurrences align with the reported and projected increase in CVD-related deaths in sub-Saharan Africa^31,32^.

However, our results showed a relative decrease in the prevalence of CA and LV atherosclerosis between the 2010–2014 and 2015–2019 time periods for most population groups, with only females of African descent having a slight increase in LV atherosclerosis, suggesting that atherosclerosis-related deaths were not the main driving force behind the rise in circulatory system disease-related deaths in our region. Interestingly, in the Western Cape Province, ischaemic heart disease increased from the fourth (5.8%) to the second (6.1%) leading underlying cause of natural death from 2010 to 2018. Conversely, in the Free State Province, ischaemic heart disease went from the tenth (1.8%) leading underlying cause of natural death to falling off the list of leading causes in the province during this period. During the same time, hypertensive diseases in the Free State increased from the eighth (2.6%) to the number one (6.0%) cause of natural deaths in the province. Yet, it stayed constant in the Western Cape as the ninth leading natural cause of death (3.3 and 3.8%). Cerebrovascular diseases increased slightly between 2010 (4.3%) and 2018 (5.1%) but dropped from fifth to sixth on the list of leading causes of natural deaths in the Free State. However, other forms of heart disease decreased in the same period from 5.3% to 4.5% and went down from fourth to seventh on this list. In the Western Cape, other forms of heart disease increased slightly (3.2 to 3.3%) but remained the tenth leading cause of natural death in the province^3,33^. We believe that the distribution of ischaemic and other forms of heart disease further strengthen our findings in our population and provide a plausible explanation for the divergent results we found in relation to the Western Cape study by Van Kets et al.^22^.

Modifiable risk factors for atherosclerosis, such as tobacco use, hypertension, hypercholesterolaemia, obesity, HIV infection and diabetes mellitus, have been identified. In high-income countries a significant decline has been observed in vascular-disease mortality, mainly due to drastic changes in public health behaviors and the improved treatment protocols for these risk factors. However, in order for these successes to continue, and to be replicated in lower income regions, increased efforts are vital to address and manage these major risk factors^12^.

Unfortunately, one of the limitations of our study was that the presence of other CVD risk factors were poorly and inconsistently reported in our current study. The socioeconomic status of the deceased is also not captured during forensic post-mortems. Therefore, finding a possible reason for the decreased prevalence of atherosclerosis in our region is challenging. The descriptive nature of this post-mortem study is a further limitation, and without significantly more data available, any conclusions made regarding contributing factors will be speculative at most. Thus, we recommend more extensive prospective studies in the same setting to capture additional important relevant data for our population group.

However, we believe our results are vital to help inform and influence public health policies in our region and contribute to more concentrated efforts to help identify target areas in specific age, gender and ethnic groups that may require enhanced public health interventions.

## Conclusion

Our results confirm the diverse CVD epidemiological profiles across different age, gender and ethnic groups in our region. The substantially lower prevalence of atherosclerosis in the African descent group across genders and age groups shows a direct correlation to the lower incidence of CVD reported in the African descent population in South Africa^8^. The European descent population in the Free State showed a very high prevalence of atherosclerosis throughout the study; however, a considerable decline in prevalence was observed from 2010–2014 to 2015–2019, indicating that this group has a similar declining trend in CVD to what is observed in high-income countries. We believe that our findings show that CVD aetiology is a multidirectional, multifaceted and dynamic process. Thus, it is imperative to perform more in-depth epidemiological studies in our population to fully understand cardiovascular health in our region. We trust that our findings may help in identifying specific groups with a potential high-risk for CVD, and can contribute in inspiring more focussed research to determine potential high-risk behaviours within specific high-risk groups, that my ultimately result in improved public health policies and awareness campaigns in our region.

## Data Availability

The datasets generated during and/or analysed during the current study are available from the corresponding author on reasonable request.
